# Parotid gland solitary fibrous tumor with mandibular bone destruction and aggressive behavior

**DOI:** 10.4317/jced.51256

**Published:** 2014-07-01

**Authors:** Estefanía Alonso-Rodríguez, Teresa González-Otero, Alejandro Castro-Calvo, Elena Ruiz-Bravo, Miguel Burgueño

**Affiliations:** 1 MD. Department of Oral and Maxillofacial Surgery, Hospital Universitario La Paz, Madrid, Spain; 2 MD. Department of Otorhinolaryngology, Hospital Universitario La Paz, Madrid, Spain; 3 MD. Department of Pathology, Hospital Universitario La Paz, Madrid, Spain

## Abstract

Introduction: Solitary fibrous tumor is associated with serosal surfaces. Location in the salivary glands is extremely unusual. Extrathoracic tumors have an excellent prognosis associated with their benign clinical behavior. We report an aggressive and recurrent case of this tumor. We review the clinical presentation, inmunohistochemical profiles and therapeutic approaches. Case Report: A 73-years-old woman presented a mass in her right parotid gland. She had a past history of right superficial parotidectomy due to a neurilemoma. FNAB and magnetic resonance were non-specific. After a tumor resection, microscopic findings were spindled tumor cells with reactivity to CD34, bcl-2 and CD99 and the tumor was diagnosed as Solitary Fibrous Tumor. The patient suffered two recurrences and the tumor had a histological aggressive behavior and a destruction of the cortical bone of the mandible adjacent to the mass. A marginal mandibulectomy with an alveolar inferior nerve lateralization was performed. Conclusions: Solitary fibrous tumor is a very rare tumor. Usually, they are benign, but occasionally they can be aggressive. Complete resection is the most important prognostic factor and no evidence supports the efficacy of any therapy different to surgery. Due to the unknown prognosis and to the small number of cases reported, a long-term follow-up is guaranteed.

** Key words:**Solitary fibrous tumor, parotid mass, parotid gland, salivary gland, rare tumors.

## Introduction

Solitary fibrous tumor was first described in the pleura by Lietaud in 1767, followed by Wagner in 1870. Klemperer and Rabin, in 1931, classified pleural tumors into two types: diffuse mesotheliomas and localized mesotheliomas or solitary fibrous tumor [SFT].

SFT is one generally associated with serosal surfaces, but in recent years it has been reported in extrapleural sites such as the liver, adrenal gland, skin, and less commonly in the head and neck, accounting for around 6% of the cases (1). In 1991, Witkin and Rosai (2) were the first to report a case in the head and neck region. A SFT located within the salivary glands is very unusual and with few cases described in the literature (3-9).

These tumors tend to present as a well defined, palpable and slowly growing masses. Clinical presentation and radiographic findings are similar to that of other tumors which are more common in salivary glands.

Extrathoracic SFT has an excellent prognosis associated with their benign clinical behavior. We report an atypical and recurrent case of SFT with aggressive behavior. This article reviews the clinical presentation, histologic features, inmunohistochemical profiles and therapeutic approaches of SFT of the parotid gland in the context of a case report.

## Case Report

A 73-year-old woman presented with a several months history of a mass in the right parotid gland region. She had a past medical history of right superficial parotidectomy due to the presence of a neurilemoma [cellular schwannoma] which had been treated 17 years previously in another medical center. She had hypertension and hypertriglyceridemia, but no toxic habits.

**Figure 1:**
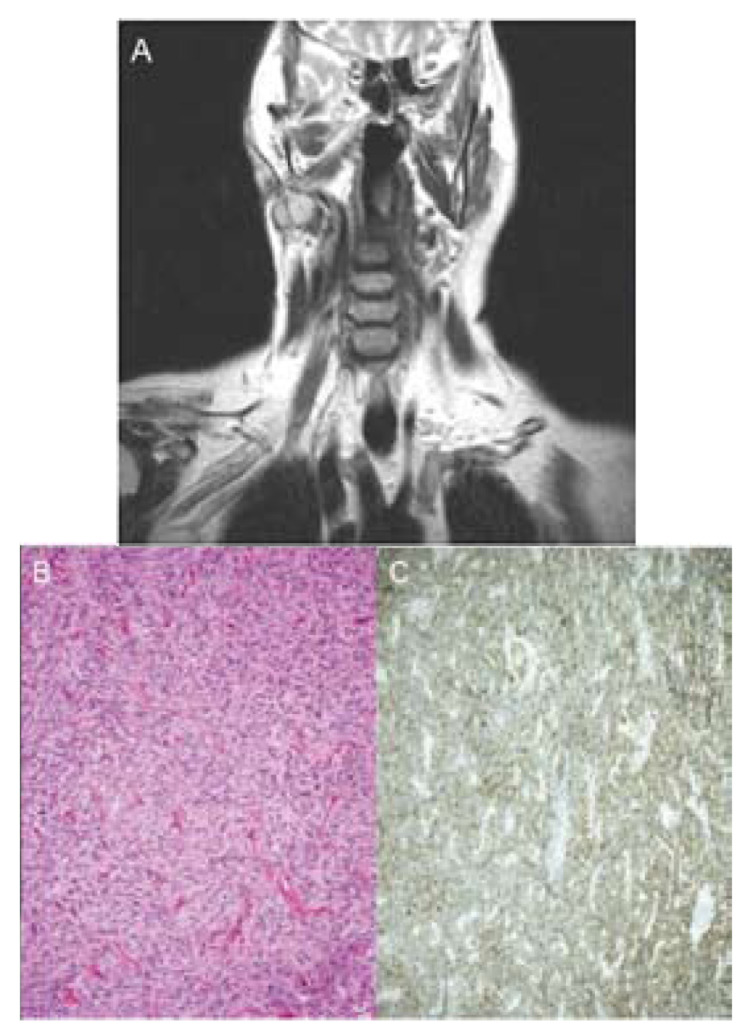
A. Contrast-enhanced T2-weighted magnetic resonance image. Note a well-defined tumor in the right parotid gland. It shows high signal intensity with homogeneous enhancement; B. Tumor cells arranged in a storiform pattern. C. They show reactivity with bcl-2.

She presented with a 2 cm palpable, firm, mobile, painless and slowly growing mass in her right parotid gland. There was no facial paralysis or constitutional symptoms and no palpable nodes on neck examination. FNAB was unspecific. Magnetic resonance imaging revealed a well-defined tumor measuring 2 cm in its greatest dimension, in the supposed location of the superficial lobe of the right parotid gland. It showed high signal intensity with homogeneous enhancement in T2-weighted images (Fig. 1). The principal suspicion was a recurrence of parotid schwannoma.

The patient underwent a tumor resection. The specimen was well-defined and not encapsulated, measuring 2 x 2 cm with a white surface. Microscopic findings were that of tumor cells arranged in a storiform pattern with numerous ramifying vessels and hyalinized walls (Fig. 1). The tumor cells were round to spindled, with tapering cytoplasm. These cells exhibited a characteristic inmunophenotype. They showed reactivity to CD34, bcl-2 (Fig. 1) and CD99, but not to AE/1, AE/2, EMA, or S-100. The tumor exhibited a Ki67 index of 5%. Based on these findings, the tumor was diagnosed as SFT.

Two years later, the patient suffered a recurrence. She developed a mass measuring 6 cm localized under the masseter muscle. During the excision of the tumor, destruction of the adjacent mandibular cortical bone was observed. The margin of the tumoral resection in this location was microscopically positive.

**Figure 2:**
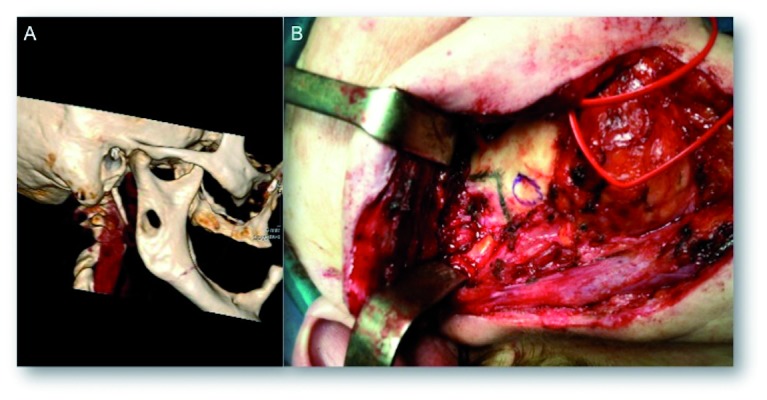
A. 3D CT image showing osteolytic lesion; B. Intraoperative view showing destruction of the buccal cortical bone of the mandible produced by the tumor.

A new magnetic resonance imaging and a computed tomography were carried out (Fig. 2). An heterogeneous image within the masseter muscle was observed which was compatible with possible tumoral remains. There was an osteolytic lesion in the ramus of the mandible suggestive of invasion of the mandibular nerve foramen (Fig. 2).

**Figure 3:**
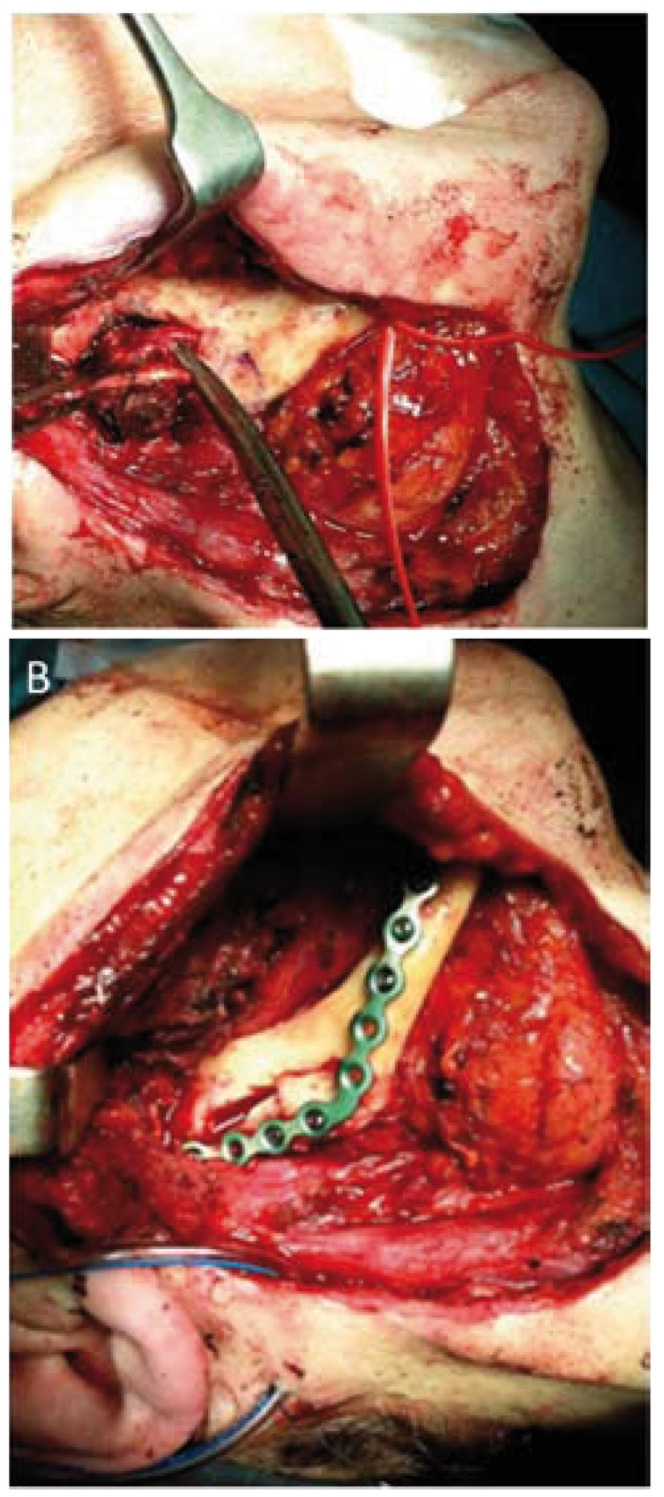
Intraoperative views. A. Mandibulectomy with an alveolar inferior nerve lateralization; B. Bone grafts from mandibular angle and a reconstruction plate for reconstructing the defect.

In this context, a marginal mandibulectomy with an alveolar inferior nerve lateralization was performed (Fig. 3). We confirmed negative margins of the resection in the bone, the nerve and the residual masseter muscle. We used bone grafts from the mandibular angle and a reconstruction plate to provide better stability (Fig. 3).

The clinical aggressiveness of bone destruction correlated histologically with an increased in Ki67 index [15%] and mitotic rate. Microscopically, bone remodelling without infiltration was observed. The patient has remained free of disease for 9 months postoperatively.

## Discussion

SFT is an uncommon benign lesion of mesenchymal origin. The finding of SFT in the head and neck is rare. Moreover, very few cases affecting the partotid gland have been described in the literature. There is no predilection based on age or gender. Bauer *et al*. (10) presented a review of the literature extending from 1960 to 2011, finding only 22 reported cases in the parotid region, 3 of which were malignant.

These tumors usually present as a well defined, palpable, painless and slowly growing mass. Occasionally, they can produce compression symptoms which should prompt suspicion of extension to the parapharyngeal space (3). Clinical presentation is similar to that of other tumors such as pleomorphic adenomas, schwannomas, fibrous histiocytomas and lipomas. Radiographic findings are nonspecific. Magnetic resonance imaging usually shows an isointense mass on T1-weighted images and high signal intensity with enhancement in T2-weighted images (8), as was found in the case we present.

Therefore, a diagnosis of SFT can only be performed based on histological and immunohistochemical findings. The macroscopic examination of the tumor shows a well-defined and encapsulated mass which may be accompanied by bone destruction, though it is normally free of infiltration. The presence of bone destruction should prompt a suspicion of malignancy, though this finding is not an indispensable requisite as a malignant tumor can have no bone erosion. Bone destruction can be attributed to a long-standing pressure effect (11). In addition to infiltration and disease recurrence, other features of histological malignancy such as a high mitotic rate, high cellularity, pleomorphism, necrosis and hemorrhages should be assessed. In the present case, necrosis and hemorrhages were absent, but mitotic rate and Ki67 index were increased. These histological findings correlated with an aggressive clinical behavior with bone destruction and disease recurrence.

Microscopic findings of SFT include tumor cells arranged in a storiform pattern with numerous ramifying vessels and hyalinized walls. There are alternating zones of hypercellularity and hypocellularity in fibrous areas. Tumor cells are round to spindled with tapering cytoplasm. Histologically, a diagnosis of SFT is also difficult because there are many tumors that display similar findings.

For this reason, immunohistochemical examinations are required in order to make a differential diagnosis. SFT shows reactivity to CD34, bcl-2, vimentin and CD99, but is negative to keratin, EMA, S-100, desmin, smooth muscle actin and muscle-specific actin. The presence of EMA, any keratin or S-100 protein will rule out many common salivary gland tumors such as pleomorphic adenoma. CD34 is a hematopoietic progenitor cell antigen that is strongly positive in most cases of SFT, though it may not be positive in all cases (12). CD34 tends to reduce reactivity in malignant SFT when compared to benign tumors (13). Peripheral neural sheath tumors such as schwannomas are positive to CD34 in some cases, but are strongly reactive to S-100 protein. Schwannoma contain Antoni A and B areas which are absent in SFT. This was essential in arriving at a differential diagnosis in the case we present. Dermatofibrosarcoma protuberans can display CD34 reactivity but the pattern of growth and collagen deposition is different and shows more mitotic activity. There is a clinical, radiographic and immunohistochemical overlap between SFT and hemangiopericytoma. Currently, this tumor is considered a subtype of SFT. A spindle cell lipoma with a prominent spindle cell component is very difficult to distinguish. The majority of carcinomas, melanomas and lymphomas are negative for CD34.

Complete local surgical excision with negative microscopic margins is the single most important prognostic factor in SFT. Some authors have documented disease recurrence at other extrapleural sites decades after the primary tumor was first identified (1) and for this reason close long-term follow up is recommended. Surgery is recognized as the treatment of choice but adjuvant treatments such as radiotherapy or chemotherapy have also been advocated. To this date, the available literature only describes three cases of SFT that have been considered as malignant. Suarez Roa *et al*. (14) report a case in the parotid region with no anomalies at glandular level. They considered it to be a low-grade malignancy and treated it with surgery and radiotherapy. A year later, there was no sign of disease recurrence. Yang *et al*. (15) also added radiotherapy as a complementary treatment in a case of histologically malignant parotid region SFT. Messa-Botero *et al*. (7) present the first documented case of parotid SFT with distant metastases. In this latter case, resection of the primary tumor was the sole treatment administered.

Ganly *et al*. (11) suggest that patients with positive surgical margins or whose tumors have a malignant component benefit from adjuvant postoperative radiotherapy. However, there are few reports concerning this issue and their follow up periods are of short duration.

In the case we presented, there were both clinical and histological indications of aggressiveness. However, in the end we were able to successfully achieve negative microscopic surgical margins. We presented the case to the Head and Neck Oncology Committee and decided that no adjuvant therapy was necessary.

The prognosis of this tumor is generally unknown. Most extrathoracic SFT have an excellent prognosis, but some authors have reported cases with aggressive behaviour and multiple recurrences, invasion of local tissues, as well the presence of distant metastases (7,14).
